# The Essence of Lipoproteins in Cardiovascular Health and Diseases Treated by Photodynamic Therapy

**DOI:** 10.3390/biomedicines12050961

**Published:** 2024-04-26

**Authors:** Piotr Wańczura, David Aebisher, Mateusz A. Iwański, Angelika Myśliwiec, Klaudia Dynarowicz, Dorota Bartusik-Aebisher

**Affiliations:** 1 Department of Cardiology, Medical College of the University of Rzeszów, 35-310 Rzeszów, Poland; 2 Department of Photomedicine and Physical Chemistry, Medical College of the University of Rzeszów, 35-310 Rzeszów, Poland; 3 English Division Science Club, Medical College of the University of Rzeszów, 35-310 Rzeszów, Poland; 4 Center for Innovative Research in Medical and Natural Sciences, Medical College of the University of Rzeszów, 35-310 Rzeszów, Poland; amysliwiec@ur.edu.pl (A.M.); kdynarowicz@ur.edu.pl (K.D.); 5 Department of Biochemistry and General Chemistry, Medical College of the University of Rzeszów, 35-310 Rzeszów, Poland; dbartusikaebisher@ur.edu.pl

**Keywords:** cardiovascular diseases, lipoproteins, PDT therapy, HDL

## Abstract

Lipids, together with lipoprotein particles, are the cause of atherosclerosis, which is a pathology of the cardiovascular system. In addition, it affects inflammatory processes and affects the vessels and heart. In pharmaceutical answer to this, statins are considered a first-stage treatment method to block cholesterol synthesis. Many times, additional drugs are also used with this method to lower lipid concentrations in order to achieve certain values of low-density lipoprotein (LDL) cholesterol. Recent advances in photodynamic therapy (PDT) as a new cancer treatment have gained the therapy much attention as a minimally invasive and highly selective method. Photodynamic therapy has been proven more effective than chemotherapy, radiotherapy, and immunotherapy alone in numerous studies. Consequently, photodynamic therapy research has expanded in many fields of medicine due to its increased therapeutic effects and reduced side effects. Currently, PDT is the most commonly used therapy for treating age-related macular degeneration, as well as inflammatory diseases, and skin infections. The effectiveness of photodynamic therapy against a number of pathogens has also been demonstrated in various studies. Also, PDT has been used in the treatment of cardiovascular diseases, such as atherosclerosis and hyperplasia of the arterial intima. This review evaluates the effectiveness and usefulness of photodynamic therapy in cardiovascular diseases. According to the analysis, photodynamic therapy is a promising approach for treating cardiovascular diseases and may lead to new clinical trials and management standards. Our review addresses the used therapeutic strategies and also describes new therapeutic strategies to reduce the cardiovascular burden that is induced by lipids.

## 1. Introduction

Cardiovascular diseases (CVDs) are the leading cause of illness and death in the world, including ischemic heart disease and stroke. CVDs are the most common cause of death worldwide, accounting for about 18.6 million deaths in 2019 [1]. The most common form is non-coronary heart disease, which can lead to acute myocardial infarction (AMI) [2]. The literature confirms that less than 11% of patients survive out-of-hospital cardiac arrest [3]. Patients who have undergone a myocardial infarction require a cycle of screening, ongoing preventive care, and also coordinated follow-up visits, as there is an increased risk of developing heart failure (HF) [4,5]. Among cardiovascular diseases, the leading causes of mortality are non-coronary heart disease and stroke, which are clinical manifestations of atherosclerosis, responsible for 84.9% of deaths from cardiovascular causes [6]. During atherogenesis, lipids accumulate in the vessel wall and trigger inflammatory reactions that stimulate the progression of atherosclerosis [7]. Traditional risk factors for atherosclerosis include smoking, hypertension, diabetes, obesity, and modified lipid metabolism [8].

As early as the early 20th century, lipids began to be thought of as a key element in the formation of atherosclerotic plaques. The first studies in this regard were those by Windaus [9] and Anitschkow [10]. The results of these studies showed that cholesterol, a component of atherosclerotic plaques, is a cause in the pathogenesis of atherosclerosis. Then, 25 years later, Mueller described an increased risk of cardiovascular disease in patients who have familial hypercholesterolemia [11]. Further studies in the epidemiological field have shown a direct link between high blood cholesterol levels and cardiovascular events [12,13,14]. Only subsequent studies illustrated that the correlation of high cholesterol levels with increased risk of cardiovascular events is related primarily to low-density lipoprotein (LDL) cholesterol (LDL-C) [15,16]. However, high-density lipoprotein (HDL) cholesterol (HDL-C) is inversely correlated with mortality from ischemic heart disease [15,16]. Consequently, the “cholesterol hypothesis” was initiated, stating that LDL-C is the cause of the development of atherosclerosis. Thus, lowering LDL-C reduces the risk of myocardial infarction and also other cardiovascular events. The aforementioned hypothesis, as well as the elucidation of the cholesterol synthesis pathway, is a key moment in the beginning of a new era of drugs that targeted lipid lowering. Statins, which block cholesterol synthesis, represent the first stage of therapy due to their effectiveness in reducing LDL-C. It should be noted, however, that patients with insufficient LDL-C lowering who have been treated with statins or have statin intolerance must have additional or alter-native treatment options. One recent study suggests that there is a need to expand attention beyond LDL-C and look more closely at lipoprotein A (LpA), triglycerides, and fatty acids [17].

Lipids are regulators of biological processes that are closely related to normal cellular function, metabolism, and distribution. Any change in the components of lipids has a major impact on cellular function, the immune system, or the inflammatory response [17]. As of today, the mechanisms that characterize different types of lipids as inducers or protectors of atherosclerosis and CMD are not well studied. So, there is a need to develop new and also more reliable strategies that are strictly therapeutically targeted. 

Thanks to new tools and information technologies, it is possible to analyze whole lipid profiles in biological fluids and tissues [18,19]. Studying and defining individual lipid characteristics, including composition and abundance in biological samples, can provide a unique tool for understanding lipid disease mechanisms. In recent years, large-scale technologies based on MS/MS have begun to be applied to purified lipoprotein fractions, with the goal of discovering specific lipoprotein protein payloads for cardiovascular disease and beyond [20]. Other studies have also applied shotgun proteomics, using it to analyze LDL and HDL in plasma to identify novel lipoprotein-associated proteins. In addition, researchers have demonstrated specific signatures in patients with atherosclerosis and different types of carotid plaques [20,21]. Improved imaging techniques have provided the ability to routinely characterize as well as detect features of carotid plaque vulnerability and consequently provide predictive information about asymptomatic and symptomatic carotid artery stenosis [22,23,24,25].

**Figure 1:**
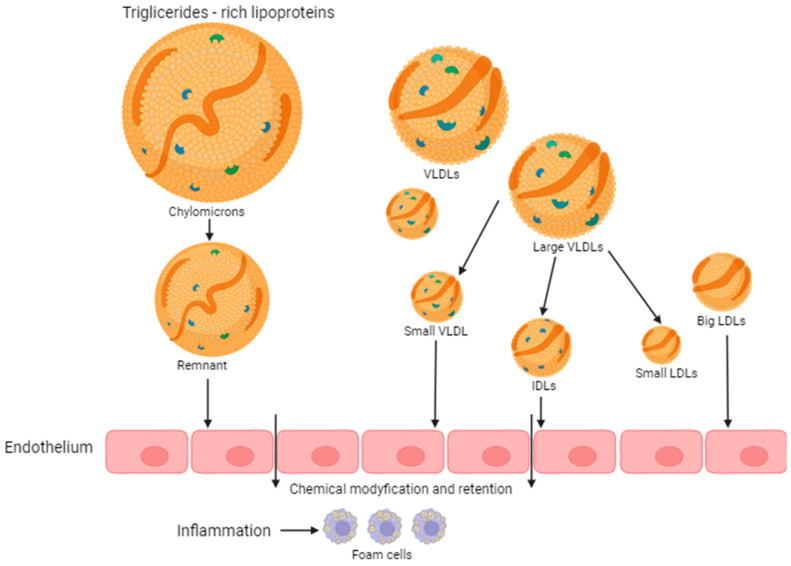
Putative mechanisms of atherogenesis involving lipoproteins. VLDL accumulation promotes pro-atherogenic changes in other plasma lipoproteins (high-density lipoprotein [HDL] and LDL) by accelerating neutral lipid exchange reactions.

These studies have been quite significant with regard to the numerous functions of each class of lipoproteins in the area of cardiovascular disease; however, to date, there is a lack of more accurate information that will provide the opportunity to assess the risk for acute clinical events. A large number of biological functions of lipoproteins, especially HDL, are based on protein and lipid components, in which changes are responsible for dysfunctional particles [26]. Researchers Castillo-Núñez and colleagues analyzed the mechanisms that contribute to the formation of atherosclerotic plaques. TRL atherogenic triglycerides cause inflammation in the arterial wall. Thus, the mechanisms are based on the generation of atherosclerotic changes in high- and low-density lipoproteins, and the accumulation of TRLs in the plasma causes them to predeposit into the subendothelial space, and there, in turn, endothelial dysfunction and vascular inflammation are generated. In addition, the accumulation of TRLs in plasma contributes to excessive viscosity and a procoagulant state (Figure 1) [27]. TRLs are a potential target for the prevention and treatment of atherosclerosis. TRL cholesterol accumulates in the plaques, and TRL triglycerides exacerbate the inflammatory component of the disease. LDL particles are separated into two main classes: large, triglyceride-rich VLDL particles (50–80 nm in diameter and 70% triglyceride mass), called VLDL1, and smaller, denser particles (30–50 nm in diameter and 30% mass of triglycerides), called VLDL2.

Currently, studies in the field of plasma lipidomics are being discussed more and more due to the fact that several pieces of evidence are emerging that indicate an association between certain types of plasma lipids, atherosclerosis, and adverse clinical events [28,29,30,31,32,33,34,35,36]. To date, there are only a few studies described that address the relationship between biologically active lipids, closely linked to their lipoprotein carriers, and cardiovascular disease [37]. A large number of studies have referred to HDL and indicated changes in phospho- and sphingolipidomes in diabetes, obesity, metabolic syndrome, dyslipidemia, and also in experimental atherosclerosis [38,39,40,41,42,43,44,45]. The normalization of the HDL lipidome in metabolic syndrome is observed either after treatment with pitavastatin or as a result of weight loss [46,47]. Changes and minimization of cardiovascular risk in the LDL lipidome have been obtained during treatment with statins or after dietary supplementation with phytosterols and omega-3 fatty acids [48,49]. There have also been analyses of HDL and LDL lipidomes in coronary artery disease (CAD) and also in acute coronary syndrome (ACS) [50,51].

Due to this, many efforts are made to discover new and more effective mechanisms for preventing and treating cardiovascular diseases. A large number of drug targets have been discovered through animal studies in the past few years. Several of these have led to significant breakthroughs in the development of new therapeutic strategies, such as those for atherosclerosis, hypertension, heart failure, and arrhythmias. There is still much work to be done since only a few of these promising solutions have been clinically used. As a result of the amazing developments of PDT therapy, we discuss its potential applications in different cardiovascular conditions in this review [52].

The following is a description of lipoprotein metabolism and an explanation of the various elements involved in this process. Each of them plays a key role in the functioning of the cardiovascular system. Before the summary is a chapter that deals with new insights into the prevention and treatment of CVDs including the use of PDT as a promising method for both prevention and treatment. 

## 2. Materials and Methods

The purpose of this review is to demonstrate the role of lipoproteins and lipids in the mechanism of cardiovascular diseases and the application of new methods of their prevention and treatment including photodynamic therapy as a therapeutic method. The FDA, PubMed, ScienceDirect, and Google Scholar scientific databases were used for this study, based on English-language data published over the past decade and beyond, using the following keywords: lipoproteins, lipids, photodynamic therapy, cardiovascular diseases, treatment. The titles and abstracts of 210 articles were reviewed.

The data used in this review came from an online search of the National Library of Medicine using PubMed from January 2012 to June 2024. This review focused on four aspects of cardiovascular (CV) diseases: the role of lipoproteins in these conditions, atherosclerosis, new methods of the treatment of cardiovascular diseases, and PDT in CVs. The following search terms were used; “role of lipoproteins in CVs” or “atherosclerosis vs. lipoproteins” or “ “lipoprotein metabolism” or “PCSK9” or “ODYSSEY Clinical Trials” or “Fatty Acid Amide Hydrolase in LDL Metabolism” or “Treatment of Cardiovascular Diseases” or photodynamic nanosystems or “photodynamic therapy in atherosclerosis” or “New therapies in CV”.

These initial searches identified 4608 publications. Based on our inclusion and exclusion criteria, the initial 4608 publications were reduced to 210 publications used. Table 1 showed inclusion and exclusion criteria.

## 3. Results

In the small and medium arteries, cholesterol-laden macrophages accumulate in the walls and cause atherosclerosis, a chronic inflammatory disease caused by lipid metabolism imbalances and immunological responses [53]. Macrophage-derived foam cell autophagy plays an important role in lipid metabolism and cholesterol homeostasis. Atherosclerotic plaque is composed primarily of foam cells formed by macrophage internalization of modified low-density lipoprotein (LDL) [54]. Atherosclerotic plaque progression and instability are caused by this cell type, leading to myocardial infarctions (MIs) and strokes. Therefore, a promising strategy for treating atherosclerosis is to reduce lipid deposition in macrophage-derived foam cells [53,55].

### 3.1. LDL Metabolism

**Figure 2:**
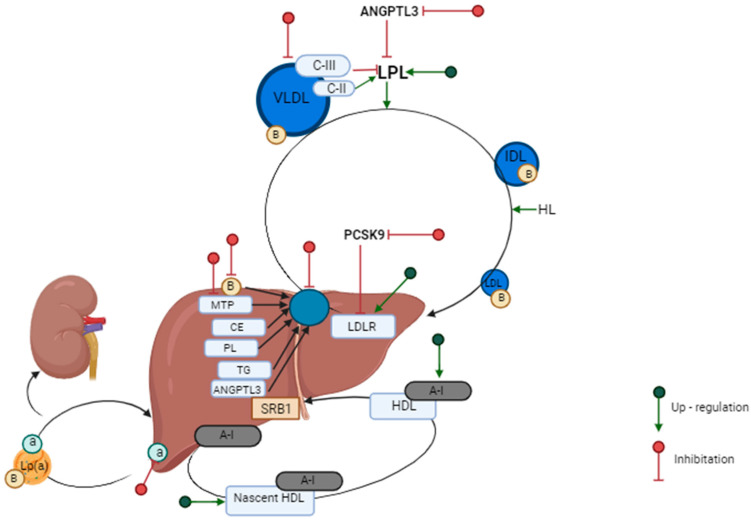
Lipoprotein metabolism, the process by which hydrophobic lipids, namely triglycerides and cholesterol, are transported within the interstitial fluid and plasma. *a*, *A-I*, *B*, *C-II*, *C-III*—apolipoprotein, *ANGPTL3*—protein that is similar to angiopoietin 3; *CE*—cholesterol ester; *HDL*—lipoprotein, characterized by high density; *HL*—liver lipase; *LP(a)*—lipoprotein (a); *IDL*—lipoprotein, characterized by medium density; *LDL*—lipoprotein characterized by low density; *LDLR*—low-density lipoprotein receptor; *LPL*—lipoprotein lipase; *MTP*—a microsomal protein that transports triglycerides; *PCSK9*—pro-protein convertase subtilisin/kexin type 9; *PL*—phospholipid; *SRB1*—receptor; *TG*—triglycerides; *VLDL*—lipoprotein that has a very low density.

The process of lipoprotein metabolism and new targets for hypolipemic therapy are shown in Figure 2. Lipoproteins are compounds that are secreted by the liver and small intestine. These compounds, lipoproteins, are complex molecules with a central core made of triglycerides and cholesterol esters. All fatty acids that are cleaved from triglycerides can be used for energy storage and/or production, and cholesterol is critical for steroid synthesis, cellular membrane formation, and bile acids. Surrounding this core is a mix of phospholipids, free cholesterol, and apolipoproteins (apos). The primary function of lipoproteins is to deliver lipids, namely cholesterol, triglycerides [TGs], and phospholipids [PLs], to peripheral tissues. The lipids and cholesterol then return to the liver (Figure 2).

Cholesterol and triglycerides are in the liver with specific apolipoproteins in very-low-density lipoprotein (VLDL) particles. Their role is to transport lipids via the blood to adipose tissue and also muscle. They are needed for energy storage and production [56]. The extraction of triglycerides from VLDL converts VLDL into LDL, that is, a lipoprotein rich in free and also esterified cholesterol, while the main lipoprotein is ApoB-100. LDL then carries cholesterol to peripheral tissues and, together with VLDL and chylomicrons, also accumulates in the vascular wall under a dysfunctional endothelium, which is the initiating and also important stage in the formation of myocardial lesions. Consequently, this results in the impairment of endothelial barrier integrity and at the same time the release of proinflammatory cytokines, chemokines, and reactive oxygen species (ROS). In addition, the recruitment of proinflammatory leukocytes and adhesion along with subendothelial transmigration are also observed [7]. In the vessel walls, macrophages that are derived from monocytes internalize LDL and then transport it to lysosomes. There, lipase hydrolyzes cholesterol esters to free cholesterol, and it undergoes esterification again by acyl-CoA/cholesterol acyltransferase (ACAT) in the endoplasmic reticulum region. This cholesterol is stored in the cytoplasmic lipid droplets, and there, it undergoes a cycle of hydrolysis and successive re-esterification. The process of removing cytoplasmic cholesterol esters is in terms of hydrolysis to free cholesterol before it is transported to the cell membrane [57]. Cholesterol removal is then initiated by several mechanisms, including the interaction of HDL with the receptor, which is a member of the ATP-binding cassette 1 (ABCA1) subfamily [58,59,60].

#### PCSK9 and ODYSSEY Clinical Trials

PCSK9 has an important function in the regulation of LDL-C homeostasis, as it binds to LDLRs on the surface of hepatocytes and also promotes their lysosomal degradation. Accordingly, PCSK9 has an effect on reducing LDL uptake, leading to an increase in LDL concentration [61]. This confirmed that PCSK9 is an attractive target for lowering LDL-C levels and thus reducing the risk of ischemic heart disease. Currently, two PCSK9 monoclonal antibodies, alirocumab and evolocumab, which are administered subcutaneously (SC) over two weeks or monthly, are being analyzed in phase III trials involving a large patient population. By sequestering PCSK9, the inhibitors of this compound block the binding of PCSK9 protein to LDLR, thereby preventing LDLR catabolism. Thus, the recycling of LDLR takes place here, thereby increasing the density of receptors across the hepatocyte surface [62]. Health Canada approved evolocumab in 2015 and, in 2016, approved alirocumab for use in patients with either familial hypercholesterolemia (FH) or very high cardiovascular risk to reduce LDL-C levels whose treatment has not worked after statin therapy. The entire clinical trial program that addresses the effect of alirocumab on the occurrence of cardiovascular events in patients with acute coronary syndrome (ODY-SSEY) consists at this point of 14 phase III trials ranging from 24 to 104 weeks and additionally includes more than 23,500 patients from around the world. It is a program to further evaluate the efficacy and also assess the safety of alirocumab versus ezetimibe in different populations [63,64,65,66,67,68,69,70,71,72,73].

In line with the above, the ODYSSEY clinical trial illustrates a significant reduction in LDL-C of about 36–61% from baseline after a minimum of 24 weeks of treatment with alirocumab. In addition, it was noted that alirocumab reduced total cholesterol, ApoB, non-HDL-C, and Lp(a) without influence from the dose of baseline statin [74]. 

Currently, the efficacy of evolocumab is being studied in the phase III clinical trial of the LDL-C Reduction Program and also on the basis of cardiovascular outcomes after PCSK9 inhibition in a range of different populations (PROFICIO). It consists of 14 clinical trials involving approximately 30,000 patients [75,76,77,78,79]. In patients treated with evolocumab (monotherapy) and either a statin or ezetimibe, reductions in LDL-C of about 49–65% have been noted simultaneously with large reductions in non-HDL-C, ApoB, and Lp(a). In addition, one recent study shows that there is regression of coronary atherosclerosis after treatment with evolocumab [80]. Bococizumab has also been evaluated in the PCSK9 SPIRE 1 and SPIRE 2 clinical trials [81]. Pfizer, the company conducting trials in this area in 2016, discontinued the bococizumab clinical development program because unexpected attenuation of LDL-C lowering and higher levels of immunogenicity were observed. ODYSSEY Outcomes is the second outcomes trial with a PCSK9 inhibitor to show a reduction in LDL-C and cardiovascular endpoints.

### 3.2. Fatty Acid Amide Hydrolase in LDL Metabolism and the Effect of Monocyte-Associated MicroRNa Expression

Fatty acid amide hydrolase (FAAH) hydrolyzes several very important endogenous fatty acid amides. A study by Rajlic et al. showed that FAAH deficiency has negative effects on the heart after ischemia and also myocardial reperfusion in mice [82]. In addition, endocannabinoids are fatty acids and thus affect receptors that are involved in the process of fatty acid metabolism; their increase may have a negative effect on cardiomyocyte survival [82].

Among the first steps in the initiation of atherosclerosis progression are the recruitment and internalization of circulating monocytes into the arterial intima–media [83]. Monocytes have been identified that express microRNAs (miRNAs), which are small non-coding RNA sequences present in the genomes of various types of organisms. Moreover, they bind to the 3′ untranslated region (UTR) of specific mRNAs based on the complementarity of their sequences. Thus, miRNA binding has the ability to inhibit translation or promote mRNA degradation [84,85]. miRNAs take part in the regulation of gene expression at the post-transcriptional level. They are also molecular markers that assist in the detection of various types of diseases due to their presence in many body fluids (e.g., serum, plasma). Some miRs are present in inflammation and oxidative stress, and their dysregulation has been linked to cardiovascular disease and atherosclerotic plaque formation, among other outcomes [86].

Several of the miRs involved in inflammation and oxidative stress, processes that occur during the pathogenesis of atherosclerosis, are miR-21-5p, miR-155-5p, and miR-221-5p. MiR-21-5p is highly expressed in various types of cardiovascular cells, but in cardiovascular disease, this expression is significantly deregulated in the cardiovascular region [87]. MiR-21 is present in significant amounts in the vessel wall and is observed to respond differently to shear or mechanical stresses that are present on the vessel. According to available studies, in humans, miR-21 is expressed in predominant cell types, although it is noticed in significant amounts in podocytes, dendritic cells, and CD14+ monocytes [88]. MiR-221-3p is involved in the regulation of vascular physiological processes and in some vascular-associated pathological mechanisms [89]. However, studies in this range show a link between the 5p chain and the above miR together with reverse left ventricular remodeling, heart failure, and arterial overgrowth [90,91]. 

Another type of miR is miR-155-5p, whose expression is affected by various inflammatory signals, including lipopolysaccharides, interferon-β, tumor necrosis factor, or the atherosclerotic process [92]. According to a study, a deficiency of hematopoietic miR-155-5p in mice having hyperlipidemia increases the development of atherosclerotic plaques by making them less stable [93]. In contrast, other studies in this area included analysis of miRNA expression profiles of monocytes in which identification of differences in expression between monocyte subsets was performed [94,95]. However, the influence of miRs on monocytes in the process of atherosclerotic plaque formation is currently not well understood.

In a study by Torres-Paz and colleagues, it was shown that monocytes from patients who had coronary artery disease (CAD) significantly increased the expression of both miR-21-5p and miR-221-5p and also decreased the expression of miR-155-5p and NOS3 [96]. Moreover, only overexpression of miR-21-5p and miR-221-5p is closely associated with increased risk of coronary artery disease. Other studies have reported elevated expression of miR-21-5p in mononuclear cells and also plasma of patients having CAD [97,98]. There is a suspicion that the aforementioned miR may be involved via the regulation of inflammatory cytokines through a signaling pathway (JNK) [99] or the phosphatidylinositol 3-hydroxykinase [PI3K]/protein kinase B [AKT] pathway [100]. This could lead to the proliferation and migration of vascular smooth muscle cells, which in turn could lead to more severe vasoconstriction and worsening of CAD [101]. Based on the literature, the overexpression of miRNA-21 inhibits the expression of peroxisome proliferator-activated receptor alpha (PPARα), and this has the effect of enhancing expression in the VCAM-1 and MCP-1 bands [102]. Hydrogen peroxide and lipopolysaccharide (LPS) also differentially affect miR-21 expression in the endothelial cell area before and after co-culture with monocytes [103]. In addition, this study indicates a role for miR-21 in terms of the unfolding of an atherosclerotic-like process, but the evaluation of the impact of increased miR-21 expression in these cells in relation to atherosclerosis requires more detailed studies.

According to other studies, miR-221-5p expression is significantly higher in the plasma of patients suffering from heart failure and atherosclerotic complications [90,104]. Again, other studies that have been conducted on endothelial cells have shown the elevated expression of the 3p chain of this miR in patients having CAD [102,105]. Torres-Paz and colleagues also showed that miR-155-5p is downregulated in CAD patients [96]. According to the literature, the expression of miR-155-5p may have various effects depending on the stage of atherosclerotic plaque development. Accordingly, its downregulation may represent a feedback mechanism to control excessive immune cell activation. A study that was performed on a mouse model of atherosclerosis says that the expression of miRNA-155-5p decreases in infused cells in response to inflammation, i.e., it may be related to the process of atherosclerotic plaque formation [106].

In addition, there was also a study in mice that showed that NOS3 deficiency resulted in increased atherosclerotic plaque formation, which caused coronary artery disease along with other cardiovascular complications [107]. Cengiz and his co-workers, after conducting a study on hypertensive patients, found that miR-21-5p expression was positively correlated with cIMT and also negatively correlated with serum nitric oxide levels and plasma area NOS3 activity [97]. There are also studies that have linked miRNA-21-5p and miRNA-221-5p expression to NOS3 in animal models and humans [108,109]. MiR-21-5p is induced during hypoxia by HIF-1, which may affect NO levels [110]. Furthermore, miR-221-3p inhibits adiponectin-stimulated NOS3 phosphorylation and NO production [111]. In addition, transfected endothelial cells show an apparent decrease in miR-221 after statin treatment, which is associated with an increase in NOS3 mRNA levels [112], but another study showed that miRNA transfection in endothelial cells decreased NOS3 protein levels [113].

### 3.3. Lp(a)

Lp(a) is a lipoprotein molecule similar to LDL with an Apo(a) molecule. It is a hydrophilic glycoprotein that is covalently bound to ApoB-100 via a disulfide bond [114]. Lp(a) and its plasma levels are determined by genetic changes in the LPA gene, which encodes Apo(a). High levels of Lp(a) determine CVD risk in patients with FH and also in the general population [115]. Lp(a) is dependent on lifestyle and drug interventions [116]. Current therapeutic options, which are limited to nicotinic acid, show a steady reduction (15–25%) [117,118,119,120]. There is growing evidence that Lp(a) has an impact on cardiovascular disease, and tyrosine oligonucleotide (ASO) drugs have emerged as a promising method to reduce Lp(a) levels (clearance conditions). Animal studies have shown that Apo(a)-specific ASO significantly reduced hepatic Apo(a) mRNA expression in mouse models [121].

Lp(a) has been linked to atherogenesis through processes such as increasing endothelial cell adhesion and expression of molecules, promoting foam cell formation by binding to macrophages with high affinity, and also interfering with vascular permeability [122]. A component of Lp(a), apolipoprotein(a), shares a large number of structural similarities with plasminogen, which interferes with the physiological process of fibrinolysis and also contributes to a prothrombotic phenotype [123].

Assessing the effect of Lp(a) on CVD has been difficult, as most laboratory animals (mice, rats, rabbits) do not express endogenous Lp(a). However, transgenic mice overexpressing human apo(a) show increased atherosclerosis [124]. Thus, Lp(a) affects the inner vascular membrane and atherosclerotic lesions and, in addition, accumulates in areas where there is vascular damage [125]. The mechanisms by which Lp(a) may cause atherosclerosis and cardiovascular risk are based on the induction of adhesion molecules, which are VCAM-1, ICAM-1, and also proinflammatory chemokines (MCP-1) found in endothelial cells; foam cell formation due to Lp(a) uptake in macrophages; and proinflammatory production of IL-8 along with reduced expression of inducible nitric oxide synthase (iNOS). Lp(a) has antifibrinolytic properties and thus can inactivate the tissue factor pathway inhibitor, which is an important inhibitor of coagulation [126].

### 3.4. Inhibitory ApoC-III

ApoC-III is a glycoprotein that consists of 79 amino acids and is synthesized primarily in the liver. It binds to lipoproteins that contain ApoB or ApoA, including chylomicrons, residues of VLDL particles, intermediate-density lipoproteins (IDL), LDL, and HDL [127]. Some assays have detected ApoC-III, which is associated with Lp(a) [128]. ApoC-III is a non-independent CVD risk factor [129]. The literature says that LDL or HDL that contains ApoC-III is closely associated with a high risk of CVD events and LDL or HDL that is devoid of ApoC-III failed to predict the incidence of CVD [129]. ApoC-III is a key regulator of plasma TG metabolism and also of TG-rich lipoproteins (TRLs) [130]. In addition, ApoC-III is a known potent inhibitor of ApoC-II-based LPL activation, which is thought to inhibit hepatic lipase activity but otherwise promotes intrahepatic VLDL folding and secretion, while interfering with TRL residue turnover. 

The mechanisms from the range of action as well as the regulation of LPL-independent pathways currently have not yet been thoroughly elucidated. However, a study was conducted where three patients with <5% normal LPL activity and fasting plasma TG values (15.9–23.5 mmol/L) were treated with Volanesorsen (Akcea Therapeutics). After 13 weeks, plasma ApoC-III concentration decreased by 71–90% while TG concentration decreased by 56–86%. These studies focused primarily on familial and multifactorial chylomicronemia and various types of hypertriglyceridemia and lipodystrophy (Table 2) [130,131,132,133,134,135,136,137,138,139,140,141,142,143,144,145,146,147,148,149,150,151,152,153,154,155,156].

### 3.5. CETP

CETP is a hydrophobic glycoprotein (476 amino acids) [157]. It belongs to the family of proteins that transport lipids. It plays an important role in reverse cholesterol transport (RCT) and, more importantly, mediates the transfer of cholesterol esters (CEs) and also triglycerides (TGs) between high-density lipoprotein (HDL) and apolipoprotein B-100 (apoB-100) [158]. According to genomic studies, CETP single-nucleotide polymorphisms (SNPs) carry out modification of plasma lipid profiles as a result of altered responses to diet [159]. Accordingly, SNPs along with loss of CETP activity are associated with sub-elevated as well as decreased HDL cholesterol (HDL-C) or LDL cholesterol (LDL-C) [160]. The process of CETP inhibition has proven to be a method for reducing ASCVD-related events by manipulating plasma HDL-C and LDL-C concentrations [161,162,163].

**Figure 3:**
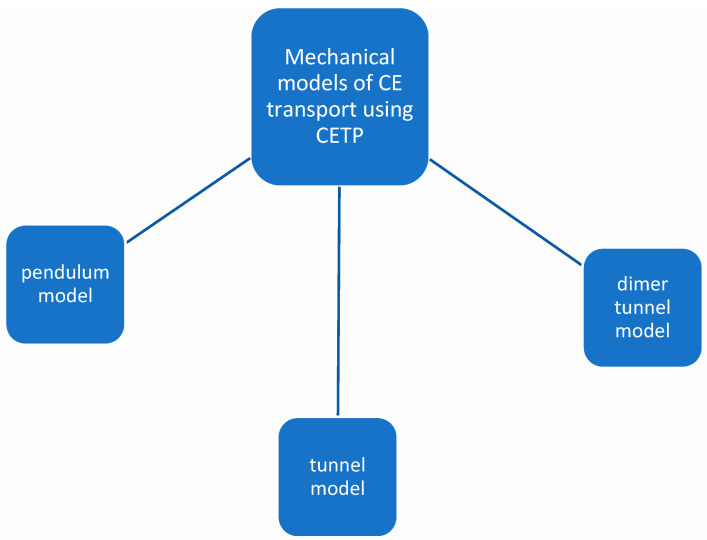
Mechanical models of CE transport using CETP.

Since the discovery of CETP, three mechanistic models of CETP-mediated CE transport have been defined (Figure 3) [164,165,166,167]. The first is the shuttle model, in which CETP binds to HDL, resulting in the deactivation of the CE molecule of HDL which consequently gives rise to the eCETP-CE complex, and this desorbs from the HDL surface and then diffuses toward LDL/VLDL. Thus, there is an exchange of CEs for glycerol lipids before returning to HDL. In this model, only binary CETP–lipoprotein complexes are formed [168]. The next model, the tunnel model, is characterized by the fact that a ternary complex, namely CETP, HDL, and LDL/VLDL, exchanges HDL-CE for VLDL-TG using a tunnel in the CETP region [169]. The last model involves a dimer tunnel, or modified tunnel model (the hydrophobic tunnel is formed by a CETP dimer instead of a monomer) [157]. These models illustrate the basic process of lipid transport, but the exact course of the mechanism is currently unknown, since lipoproteins and CETP–lipoprotein complexes have dynamic properties and additionally have structural flexibility [170,171,172,173].

CETP deficiency genetically increases the number of HDL particles, and the partial breakdown of CETP has a similar effect, which is associated with increased plasma APOA1 [174,175]. Studies conducted in heterozygous patients for CETP mutations were closely correlated with a lower number of ASCVD events as well as increased HDL-C levels [176]. These studies show that inhibition of CETP is protective against atherosclerosis, but later studies of heterozygous families with CETP deficiency found no evidence of premature atherosclerosis [174,175]. Genetic studies have also shown that CETP deficiency is an independent risk factor for ASCVD [177]. This is inconsistent with strategies to reduce ASCVD by inhibiting CETP. One analysis of the combination of three SNPs of the CETP gene showed that CETP genotypes are associated with inhibition of CETP activity as well as moderately higher HDL-C levels, suggesting that the CETP gene is poorly associated with the risk of ASCVD [178]. Conversely, the CETP gene is associated with LDL, which is mechanically linked to premature ASCVD, and HDL is an ASCVD-modifying factor [179].

### 3.6. HDL

Recent years have seen significant progress in understanding the structure of HDL particles and the relationship between composition, function, and CVD. Currently, it is recognized that human HDL is a highly heterogeneous family of lipoproteins, which differ in such characteristics as density, size, shape, lipid, and protein composition. Approximately 100 HDL-binding proteins (including apolipoproteins, enzymes, and lipid-carrying proteins) have been identified so far and are distributed differently across the HDL subpopulation [180].

It is worth noting that recent studies have proven changes in composition as well as post-translational modifications in the proteome, which is bound to HDL. In addition, it has been observed that any changes in the HDL lipidome region affect its functionality, and this affects its cardioprotective properties [181,182]. Therefore, the structural as well as functional complexity of HDL means that the measurement of HDL-C concentrations is unlikely to be a reliable marker of cardiovascular risk closely associated with HDL. In addition, routine clinical practice, which is the quantitative determination of HDL-C together with its major apolipoprotein AI (ApoA-I), requires significant improvement. Thus, the ability to remove HDL [183,184] may be a useful marker. According to recent clinical studies and genetic studies, which focused on assessing the increase in HDL-C levels following pharmacological treatment or genetic polymorphisms, it has no effect on cardiovascular events [185,186,187,188]. These studies have challenged the therapeutic value of any pharmacological intervention that increases HDL cholesterol and have initiated further, more detailed studies on the structural and functional diversity of HDL [180,181,182,183,184,185,186,187,188,189].

## 4. A New Look at the Treatment of Cardiovascular Diseases

An effective strategy for reducing intracellular lipid deposition in atherosclerosis is photodynamic therapy. PDT using upconversion fluorescent nanoparticles containing chlorin e6 (UCNPs-Ce6) significantly increases cholesterol efflux by triggering autophagy in proliferating THP-1 macrophages derived from foam cells as well as peritoneal macrophages. ROS/PI3K/Akt/mTOR signaling pathways are involved in this process in part. The use of UCNPs-Ce6-mediated PDT could therefore be effective in preventing the progression of atherosclerosis [190]. In the treatment of atherosclerosis, one of the limitations of photodynamic therapy (PDT) is that the excitation light for photosensitizers (PSs) cannot penetrate deep into tissue. Furthermore, the type of PS, the accumulation of PSs in atherosclerotic plaques, and the use of animal models have significant implications. Researchers recently developed photodynamic nanosystems, on which macrophages are cleared by PDT, to prevent atherosclerosis progression [191,192,193]. In addition, pharmacotherapy that uses biomimetic drug delivery systems can improve therapeutic efficacy in atherosclerosis due to knowledge about macrophage membranes that sequester proinflammatory cytokines for the suppression of local inflammation [194]. 

Atherosclerosis is promoted by the monocyte chemoattractant protein-1 (MCP-1), which recruits monocytes to subendothelial layers in order to promote its progression [195]. In addition, tumor necrosis factor-a (TNF-a) is an established pro-atherosclerosis factor. Increased transcytosis of LDL across endothelial cells and an increase in subendothelial retention of LDL are responsible for this [196]. Besides initiating and progressing atherosclerosis, TNF alpha influences myocardial ischemia and heart failure [197]. As a result, MCP-1 and TNF alpha can be used to measure atherosclerosis treatment response.

In the treatment of atherosclerotic plaques, chemiexcited PDT appears to be an effective solution. An NP with a photosensitizer such as monomethoxypolyethylene glycol-block-poly(L-lysine) modified with Ce6 and 3,4-DA could be synthesized and self-assembled with bis(2,4,5-trichloro-6-[pentyloxycarbonyl]phenyl)oxalate (CPPO) to produce singlet oxygen accumulated effectively in plaques. It is recommended that the whole system be cross-linked with an Fe3+–catechol complex for stabilization and magnetic resonance imaging (MRI). Moreover, Fe3–catechol cross-linked CPPO-loaded mPEG-Plys-(DA-Ce6) NPs (FeCNPs) also inhibit atherosclerosis progression by eliminating macrophages in aortic arches and abdominal aortae, as shown by T1-weighted MRI. The expression of MCP-11 and TNFα is significantly reduced after PDT treatment. Overall, CPPO-excited PDT is a promising and potential treatment for atherosclerosis via macrophage elimination, and plaque-targeting ability needs to be improved for better therapeutic efficacy [198].

Despite advances in diagnostic methods, atherosclerosis in small coronary arteries cannot yet be imaged effectively with current diagnostic methods. In order to visualize high-risk plaques that can lead to myocardial infarction, new high-resolution molecular and structural imaging strategies are necessary. Indocyanine green (ICG) is currently being used in clinical practice for imaging retinas and choroids with optical methods. Atherosclerotic plaque imaging can also be enhanced by ICG’s ability to quickly bind to lipid-rich plaques and cells [199]. Using a clinical-type intravascular guidewire in the aorta for in vivo detection of lipid-rich atherosclerotic plaques in coronary arteries, an indocyanine green (ICG) compound was found to provide sufficient signal enhancement in five rabbits with atherosclerosis. Ultrasound and angiography were used to confirm the location of these plaques. Moreover, lipid-loaded macrophages in atherosclerotic plaques were confirmed as the preferred target of ICG in an early clinical study using freshly resected carotid endarterectomy specimens from four patients. Consequently, ICG is promising as an early detection tool for atherosclerotic lesions in vessels, but it is also capable of detecting unstable atherosclerotic plaques, which are of high risk to humans [200]. 

A key limitation of photodynamic therapy (PDT) in treating atherosclerosis is that the excitation light for photosensitizers (PSs) has a low tissue penetration ability, as well as the type of PSs and their accumulation in atherosclerotic plaques, as well as the use of animal models to conduct experiments. Photodynamic nanosystems have recently been developed, aimed at stopping the progression of atherosclerosis via the clearance of macrophages by PDT [191,192,193]. Furthermore, thanks to knowledge about the properties of macrophage membranes that sequester proinflammatory cytokines to suppress local inflammation, targeted pharmacotherapy can achieve improved therapeutic efficacy in atherosclerosis using biomimetic drug delivery systems [194].

Numerous studies have shown that porphyrin-based photosensitizers accumulate selectively inside atherosclerotic plaques. An experiment in rabbits showed increased uptake selectivity as a result of preassociating a benzoporphyrin derivative with low-density lipoprotein and acetylated LDL (Ac-LDL) compared to normal arteries [201]. Another study involving rabbits and atherosclerosis found that selective accumulation of hematoporphyrin derivatives was observed throughout the plaque thickness. As a result, human atheromatous plaques should be able to take up hematoporphyrin derivatives in vivo, making photochemical treatment as an alternative to atherosclerosis a potential therapeutic approach [202]. In clinical settings, however, hematoporphyrin photosensitizers are not applicable due to cutaneous photosensitivity and poor penetration of light at 630 nm through blood vessel walls [203].

A potent second-generation photosensitizer derived from hematoporphyrin, verteporfin, is also known as benzoporphyrin derivative monoacid ring A (BPD-MA) [204]. Due to the interaction between verteporfin and endogenous low-density lipoproteins, atherosclerotic tissue accumulates more of this photosensitizer than normal arteries do. Furthermore, it facilitates the removal of atherosclerotic plaque through apoptosis when activated by light [205]. Humans, rabbits, and miniswine with Watanabe hereditary hyperlipidemia took up BPD-MA in their atherosclerotic plaques [201,206].

5-Aminolevulinic acid (5-ALA) is a precursor of protoporphyrin-IX. Photodynamic therapy with 5-ALA has been tested on rabbits and pigs for the treatment of atherosclerotic plaques [207,208]. According to these studies, 5-ALA-based PDT may be an effective method for preventing and treating atherosclerosis [209]. 

## 5. Discussion

Epidemiological and genetic studies suggest that long-term exposure to LDL-C is an important factor in atherosclerotic vascular disease. Currently, efforts are being made to lower LDL-C levels as a primary therapeutic goal aimed at preventing cardiovascular events. Statins are used here as first-stage therapy. Based on the studies conducted, it is the amount of LDL-C reduction during treatment with statins that is the main factor that reduces the risk of cardiovascular disease. However, a clinical problem is still the rather limited efficacy of the above treatment. Additional drugs that lower LDL-C (ezetimib, PCSK-9 inhibitors) minimize cardiovascular risk. This is a kind of LDL-C-lowering potential. Research on these drugs is ongoing (bempedic acid, cholesterol synthesis inhibitor, PCSK9 synthesis inhibitors). Its task is to demonstrate the importance of the cardiovascular system in relation to the potential of lowering LDL-C, and above all to demonstrate high tolerance as well as cost-effectiveness. Therefore, future studies must demonstrate risk reduction through additive processes of lowering LDL-C in order to be clinically applicable. 

Thanks to numerous studies and scientific advances, more and more treatments for dyslipidemia are now available. Particular attention is paid to the benefits of long-term lipid-lowering therapies such as statin, fibrate, or ezetimibe therapy or the use of PCSK-9 inhibitors. However, there are still many challenges in diagnosing and treating lipid disorders. The prevention of cardiovascular disease is crucial, so new therapies are being sought, and potential avenues for further research are being identified [210]. Therefore, further research on whether PDT can be used to treat atherosclerosis seems reasonable. PDT’s role in cardiovascular diseases is to selectively target the plaque without harming the normal vessel wall. The PS should be selectively taken up in plaques. However, the distribution of the photosensitizer within different compartments of a plaque is likely also very important. Based on clinical reports, it can be concluded that an important problem is the prevention and treatment of restenosis in cardiovascular diseases, for which the PDT technique can be used. Due to PDT’s continued success in inhibiting intimal hyperplasia in experimental animal models, it is currently being tested in clinical trials for vascular diseases. However, the ideal photosensitizer for photodynamic therapy in atherosclerosis remains an unresolved issue, and the use of photodynamic therapy deserves further research. PDT is one of the most promising approaches to inhibiting intimal hyperplasia.

## 6. Conclusions

New therapies that treat disorders that are closely related to lipids and the risks they carry are in clinical development. To find a critical application, they must meet the following conditions: meet specific needs, be safe, be relatively inexpensive, and be widely available. Each new treatment is developed on the basis of these goals. New therapies are mainly biological drugs, based on mechanisms, principles of action, or delivery systems. However, it should be noted that if these measures prove to be safe, effective, and available, the production costs of these measures may be expensive, which will limit their use. Therefore, the challenge of new methods that lower lipid levels will primarily be availability. The detailed mechanisms of PDT for CVDs are largely unknown due to the complex immune responses in different tissue microenvironments. Therefore, further research using advanced nanomaterials will enable the treatment of severe non-oncological diseases with improved phototherapy.

## Figures and Tables

**Table 1:** The inclusion and the exclusion criteria.

The Inclusion Criteria	The Exclusion Criteria
Metabolism and role of lipoproteins in CVDs	Results of the narrative review
Clinical and preclinical studies on the role of lipoproteins in the development of cardiac diseases	Studies on lipoproteins in other diseases
Research on genetic changes in lipoprotein metabolism	Exercise and cardiovascular protection
Mechanism of atherosclerosis formation	The role of adipose tissue in cardiovascular health and disease
In some cases, data from before January 2012 were used to put the current data in perspective	Use of PDT in other diseases or cancers
Cardiovascular health and diseases	
PDT as a new method of treating disorders of the cardiovascular system	

**Table 2:** Mechanisms of action of lipid disorder therapy.

	Mechanism	Emerging Therapy
	MTTP inhibition	Lomitapid
	ApoB inhibition	Mipomersena; (ApoB ASO)
VLDL production/secretion	ACL inhibition	Bempedic acid (ETC-1002)
	Acetyl Coenzyme A Carboxylase inhibition	Dicarboxylic acid derivative (Gemcabene)
VLDL secretion	ANGPTL3 inhibition	Ewinakumab (mAb ANGPTL3)
		ANGPTL3-GalNAc-ASO (ANGPTL3-L rx)
	ApoC-III inhibition	Volanosersen (ApoC-III ASO; ApoC-III RNAi (ALN-AC3)
Hydrolysis of VLDL and TG-rich lipoproteins	ANGPTL3 inhibition	Ewinakumab; ANGPTL3-GalNAc-ASO
	PPARα agonism	SPPARMα (K-877)
	LPL replacement	Alipogen tiparvovec gene therapy; AAV1-LPL S447X
The clearance of TG-rich lipoproteins	ANGPTL3 inhibition	Ewinakumab; ANGPTL3-GalNAc-ASO
	ApoC-III inhibition	Volanosersen; ALN-AC3
Exchange between HDL and TG lipoproteins	CETP inhibition	Inhibitor CETP (TA-8995)
	LCAT upregulation	Recombinant human LCAT protein (ACP-501)
	Upregulation of ApoA-I	Apo-I mimetic peptide; ApoA-I Milano transgene ApoA-I DNA plasmid
	HDL-mimicking infusion	HDL mimetic peptides
	HDL autotransfusion	Infusions of autologous HDL lipid-free/pre-β-enriched plasma
LDL briefing	PCSK9 inhibition	PCSK9 mAb (alirocumab, evolocumab); PCSK9 GalNAc-RNAi conjugate (ALN-PCSsc; Inclisiran); genome editing (PCSK9 CRISPR/Cas9); peptide-based anti-PCSK9 vaccine
	LDLR replacement	gene therapy AAV8-LDLR (RGX-501)
Peripheral fat metabolism	DGAT1 or DGAT2 inhibition	Inhibitor DGAT1 (pradigastat); DGAT2-ASO (DGAT2 Rx)
Production of Lp(a)	Apo(a) inhibition	Apo(a) ASO (APO(a)-L Rx)

## Data Availability

Not applicable.
